# A Path Out: Using Video Games to Reduce Prejudice Towards Refugees

**DOI:** 10.3390/bs15050583

**Published:** 2025-04-26

**Authors:** Liam Cross, Gray Atherton, Chris Stiff

**Affiliations:** 1 School of Psychology, University of Plymouth, Plymouth PL4 8AA, UK; gray.atherton@plymouth.ac.uk; 2 School of Psychology, Keele University, Staffordshire ST5 5BG, UK; c.stiff@keele.ac.uk

**Keywords:** videogames, outgroups, attitudes, pro-sociality, proteus effect, prejudice, empathy

## Abstract

Historically, research on video games has centred on their potentially adverse effects, though more recently, work has started to explore the benefits. Here, we investigate whether playing a video game portraying a refugee’s plight in escaping war-torn Syria could affect implicit and explicit attitudes towards that social group. We show that after 30 min of game play embodying a Syrian refugee, participants showed reduced prejudicial attitudes and increased empathy towards Syrian refugees compared to those playing a mechanically similar but contextually unrelated game. While implicit attitudes followed the same direction, this difference was not statistically significant. Potential theoretical underpinnings of these findings, including perspective taking, embodiment, and contact perspectives for prejudice reduction, are discussed.

## 1. Introduction

Video games are a multi-billion dollar industry, with a 2024 revenue of over USD 280 billion (Anonymous, n.d.-c). Thus, researchers are very interested in how this behemoth may influence us psychologically. Initial research on video games leant towards the negative, particularly regarding aggression. Many studies have linked violence in gaming with increased imitation in real life (Anderson & Bushman, 2001; Scharrer et al., 2018). Other studies have similarly focused on the negative aspects of gaming, suggesting links with obesity (Goodman et al., 2020), desensitisation to violence and sexist attitudes (Funk et al., 1999; Beck et al., 2012; Fox & Bailenson, 2009), though some work has drawn some of these findings into question (Cross et al., 2024b; Ferguson, 2020; Ferguson & Colwell, 2020).

In recent years, research has looked at more positive elements of gaming, showing that it can increase helping behaviour (Greitemeyer & Osswald, 2010), facilitate learning (Schaffar, 2016), and improve social relationships (Atherton & Cross, 2021). Violent video games can even decrease aggressive behaviour if played cooperatively (Velez et al., 2014). Thus, there is plenty of evidence to suggest how video games can be used to propagate positive social change, and psychological research is invested in discovering mechanisms to facilitate this. One method—the topic of this paper—is through the embodiment of an outgroup member in-game, leading to changes in attitudes and behaviour (the Porteus effect).

### 1.1. The Proteus Effect

Most games require players to steer a digital representation (called an avatar) during play. There are often options for participants to customise their avatars and create a representation of themselves (Lim & Reeves, 2009), heightening immersion (Taylor, 2002). Players may create avatars that are realistic and representative of themselves or idealised versions that have exaggerated or desired traits (Jin, 2010; Suh et al., 2011). When using an avatar, players may internalise their persona’s traits and display behaviours they believe are representative of that avatar (Yee et al., 2009).

A compelling finding in the research on customisation and avatars is that a player will often acquire the characteristics of their avatar during play. This in-game behavioural imitation is known as the Proteus effect (Yee & Bailenson, 2007; Szolin et al., 2022). For example, players who choose a male avatar in a game will often show biases and behaviours they think are typically “male” (Ratan & Sah, 2015). Similarly, players who choose Black avatars may espouse stereotypical behaviour they think is typical of that ethnicity (Hawkins et al., 2021). The Proteus effect can operate even when using imagined models—players who chose to play as a particular (fictional) race in a video game have reported that their behaviour is often shaped by the characteristics of that race (Stavropoulos et al., 2020). Evidence suggests that a greater degree of customisation creates a stronger Proteus effect (Ratan et al., 2019).

### 1.2. Theoretical Grounding for the Proteus Effect

The primary foundation of the Proteus effect is the self-perception theory. According to this idea (Bem, 1972; Valins, 1966), people infer their attitudes and appropriate behaviours by observing themselves as if they are a third party. Therefore, players who immerse themselves in a persona will act in a fashion they believe congruent with the expectancies of others for that persona. Factors which increase the sense of immersion and connection with one’s avatar such as more customisation (Ratan et al., 2019) or stronger identity with the avatar exacerbate this effect, as the avatar provides a stronger “cue” for how the player should act (Stavropoulos et al., 2020).

An alternative theoretical perspective that has been suggested is that the Proteus effect is a form of priming (Ratan et al., 2019). Here, embodying an avatar leads to the activation of schemas associated with the avatar’s characteristics, which in turn lead to schema- (and often stereotype-) congruent attitudes and behaviour. Although there is some evidence for this idea (Peña et al., 2009), other work has demonstrated that embodiment through an avatar has a much more profound effect compared with simply watching a third party, which would not be the case if simple priming were occurring. Thus, the self-perception theory seems the most persuasive explanation using the extant research (Yee et al., 2009).

### 1.3. Gaps in the Current Literature on the Proteus Effect

The extant literature on the Proteus effect tends to focus on the elicitation of avatar-congruent behaviours; that is, the player acting in the way they think their avatar would act. However, a yet unexplored application is the use of the Proteus effect in reducing prejudice.

The Proteus effect can be leveraged in this fashion by asking players to embody an avatar that is representative of a specific outgroup. In perspective-taking research, individuals are asked to imagine being a member of an outgroup, which then reduces prejudice towards that group (Vescio et al., 2003; Geisser et al., 2024; Shih et al., 2009). Using a Proteus effect paradigm, perspective taking also takes place, but is facilitated by embodying an outgroup member through a game and acting in a way they believe is congruent with that identity. Moreover, the vividity and immersion of this perspective-taking experience is greatly heightened, which has a more profound effect on attitude formation and change (Ganschow et al., 2021; Blondé & Girandola, 2016).

The Proteus effect also dovetails with the theoretical foundation of the contact hypotheses (Allport, 1954; Pettigrew & Tropp, 2006; Crisp & Turner, 2012) which suggest that collaborative endeavours with outgroup members reduce prejudice towards that outgroup as a whole. Contact is a highly effective method of reducing prejudice between groups (Achbari, 2015; Alperin et al., 2014; McKeown & Dixon, 2017); moreover, the concurrent physical presence of actors is not required as contact can work through electronic media such as video games (Stiff & Bowen, 2016; Stiff & Kedra, 2020; Vang & Fox, 2014). By playing as an outgroup member (in the form of an avatar) a player is essentially in contact with that outgroup through “being” part of that group, again theoretically lowering prejudice. In this paper, therefore, we look to examine whether the Proteus effect can be used to reduce intergroup prejudice towards a specific outgroup.

### 1.4. Outcomes

A challenge when examining prejudice is obtaining a true measure of the participants’ feelings. Many prejudice measures rely on self-reports, meaning participants may provide responses they think are appropriate rather than those reflecting their genuine attitude (Dermody et al., 2013). To combat this, researchers can also look at implicit prejudice, which taps more into unconscious bias (Hall et al., 2009). Implicit prejudice can be activated unintentionally by the mere presence of an attitude object and, therefore, can circumvent problems with social desirability (Turner et al., 2007).

The most ubiquitous method for measuring implicit attitudes is the implicit association test (IAT) (Greenwald et al., 1998). The IAT is a computerised task which measures how quickly and arcuately participants can respond to a topic-specific exemplar when it is paired with a positive word (e.g., “pretty”) compared with a negative word (e.g., “ugly”). Participants with greater prejudice towards the topic are generally slower to respond in the former situation and make more mistakes. The IAT has been an effective method of measuring implicit attitudes in a variety of challenging and transgressive topics such as perceptions of autism (Brickhill et al., 2023), disability bias (Cross et al., 2024a) aggressiveness (Parmač Kovačić et al., 2018), substance users (Benau et al., 2024), outgroup prejudice (Lynott et al., 2019), and self-harm (Velkoff & Smith, 2020).

As prejudice—both implicit and explicit—has not been examined within the Proteus effect research, it seems appropriate to include both kinds of measures to look for correspondence between them and see whether the Proteus effect can impact on either, both, or neither. Finally, empathy is the ability to understand and respond to others’ emotions (Stathi et al., 2021) and is a vital component of pro-sociality (Elliott et al., 2011). Both perspective taking and contact are linked with improved empathy towards the target group (Yu et al., 2020; Li & Edwards, 2021). Perspective taking elicits greater empathy towards an individual undergoing a painful task (Leong et al., 2015) and can direct attention away from bias-provoking components of an outgroup image (e.g., jewellery that is indicative of that outgroup) (Sherman et al., 2020). Contact with outgroup members can create empathy towards that entire group (Fuochi et al., 2020) and lead to meaningful behavioural change such as a reduction in hate speech (Soral et al., 2022) or an increased likelihood of intervening in race-hate situations (Abbott & Cameron, 2014). Therefore, we will test the game’s effect on empathy as well as implicit and explicit attitudes.

### 1.5. The Current Work

In summary, then, this paper extends the intergroup processes literature by examining whether the Proteus effect can be implemented as a method of prejudice reduction. By asking participants to take on the role of an outgroup avatar, we can create both a perspective-taking and contact situation. Accordingly, this should lead to attitude change in line with the other work in this area.

In this study, participants played the game “Path Out”, which was designed with this aim in mind by a Syrian individual who tells their story of escaping a war-torn Syria. Participants played using an avatar that is a Syrian refugee, and their attitudes towards refugees were then measured. We predicted that, through the Proteus effect, participants’ attitudes towards refugees would be more favourable after play compared with a control condition (H1).

As explained previously, a player taking on the identity of an outgroup member via their avatar should experience both perspective taking and contact with that outgroup through immersion in their character. Accordingly, that player should experience changes in empathy in a positive direction towards that outgroup. That is, participants who play a game with a Syrian refugee avatar should have more sympathy towards refugees compared with a control condition (H2).

Finally, previous work has shown that these attitude types can affect different domains. Specifically, explicit attitudes tend to correspond with more overt behaviour, whereas implicit attitudes relate to more subtle biases (Dovidio et al., 2002). Thus, in this paper, we will use both measures to fully explore these ideas and examine to what extent the Proteus effect might impact both implicit and explicit prejudice (RQ1).

## 2. Materials and Methods

This study utilised a between-participants design. The independent variable was the game played (Path Out—experimental and Metaphobia—control). The dependent variables were attitudes towards refugees measured through a range of self-report (explicit) questionnaires and an IAT. Participants were assigned to each condition semi-randomly while keeping overall N’s and gender balanced across conditions. Ethical approval was granted by Edge Hill University’s ethical review board, and all participants gave full informed consent. Seventy-six participants (38 males, 38 females, Mage = 21.5, age range 18–28) took part in total, and they were recruited from Edge Hill University using opportunity sampling and the Sona system.

Participants in the experimental condition were asked to complete the first level of Path Out, an autobiographical adventure game that allows the players to replay the journey of Abdullah Karam, a young Syrian artist who escaped the civil war in 2014 (“Path Out”) (Anonymous, n.d.-a). The game was designed to educate participants on the journey Syrian refugees took when they fled the country in 2014. It was developed in the hope that participants would better understand refugees’ journeys after playing. The first level of the game was chosen to allow for ease of play while allowing participants to be exposed to the relevant narrative. It had a varied completion time of around 30–40 min (mean = 36.40 min). A crib sheet was designed to ensure that participants could complete the task in the allotted time and overcome any sticking points. This crib sheet was designed by the researcher, noting the main sticking points the researcher and participants in the pilot study struggled with, such as the locations of tasks and reminders of items participants would need to collect.

Participants in the control group were asked to play Metaphobia (Anonymous, n.d.-b), an investigative mystery game in the style of classic 1990s point-and-click adventures. In Metaphobia, players play as Richard Elmsat, son of murdered mayor Carl Elmsat. Players play through the narrative and attempt to solve the murder case. This game was chosen because it is a similar top-down puzzle platform game, but it contains no political narrative. Participants played for 35 min to provide a similar game exposure time among each condition.

The attitudinal questionnaire was composed of questions from a prejudicial attitudes questionnaire (Hoyt & Goldin, 2016) and a directed attitudinal measure (Atherton et al., 2019; Atherton & Cross, 2020) assessing relevant stereotypes and empathy towards the target group (refugees). Participants rated their agreement on a set of questions on a Likert scale, where 1 represented “totally disagree” and 5 represented “totally agree”. The questionnaire measured two constructs, with five questions measuring empathy (“I feel empathy towards Syrian Refugees”, “I feel the same emptions as Syrian refugees”, “I relate to the emotions that a Syrian refugee may feel”, “I feel like I understand Syrian refugees and why they need to seek asylum”, “I have an understanding of what its like to walk in a Syrian refuges shoes”), and 9 attitudes (“I would happily have a Syrian refugee as my boss/sexual partner/partner in marriage”, “Syrian refugees are lazy”, “Syrian refugees are undeserving of governmental support”, “Syrian refugees should have the same rights as British people”, “Syrian refuges receive too much support”, “Syrian refugees should have access to employment as British people”, “Syrian refugees are no different to my friends and family”). Relevant items were reverse scored, so in all cases larger numbers represented more positive attitudes/greater empathy towards the target group.

Implicit attitudes were measured using an IAT (Greenwald et al., 1998), which allowed for the measurement of automatic, implicit evaluations towards refugees versus native people. The IAT measured the speed and accuracy in which participants made correct pairings between attributes (a selection of words meaning good or bad, i.e., Wonderful/Hate and a target (Refugee versus Native, i.e., Outsider/National), using the I/E keyboard keys. In half of the critical trials, participants were categorising one attribute/target pairing (Native-Good/Refugee-Bad) to one side of the screen using one key press (I/E) and vice versa, and in the other critical blocks, the reversed pairings (Refugee-Good/Native-Bad). There were two critical blocks for each pairing. The IAT gauges the strength of these associations by creating a weighted score (from −2 to +2) for the speed and accuracy of the participants making pairings—or associations—between concepts. A score closer to +2 indicated a stronger association between Native/Good, Refugee/Bad, whilst a score closer to −2 showed a stronger association between the concepts Native/Bad, Refugee/Good) (Greenwald et al., 2003).

## 3. Results

**Figure 1:**
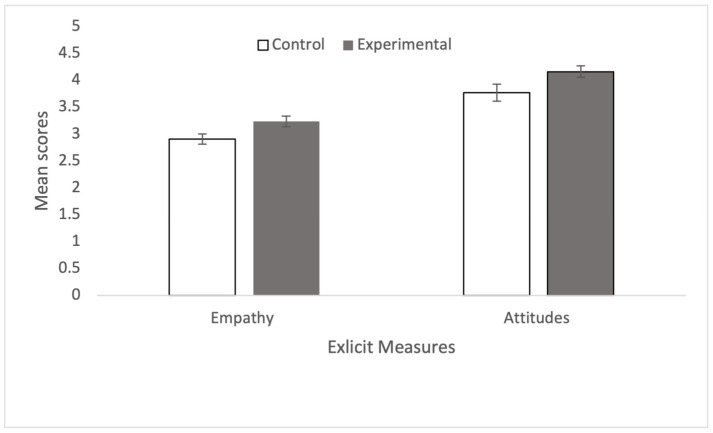
Shows the mean and standard errors for the average attitude and empathy self-report measures.

**Figure 2:**
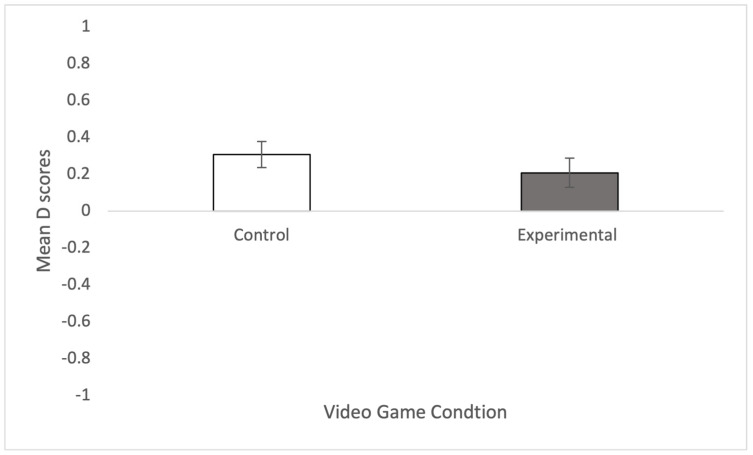
Shows the mean and standard errors of the D scores for the IAT.

A *t*-test showed that the explicit attitude scores were significantly greater amongst those in the experimental condition (M = 4.169, SD = 0.671) than those in the control condition (M = 3.777, SD = 0.972), t(74) = 2.057, *p* = 0.022, d = 0.472, supporting H1. Similarly, the empathy scores were significantly greater amongst those in the experimental condition (M = 3.240, SD = 0.623) than those in the control condition (M = 2.911, SD = 0.592), t(74) = 2.358, *p* = 0.011, d = 0.541, supporting H2. In relation to RQ1, while the IAT scores were smaller on average amongst those in the experimental condition (M = 0.209, SD = 0.490) than those in the control condition (M = 0.308, SD = 0.429), this difference was not significant, t(70) = 0.912, *p* = 0.182, d = 0.215. Figure 1 and Figure 2 show the mean and standard errors for explicit (Figure 1) and implicit (Figure 2) measures.

## 4. Discussion

In this paper, we examined to what extent the Proteus effect could afford a reduction in prejudice following relevant video gameplay. Participants played a game in which they controlled an avatar representing an outgroup. According to the Proteus effect, participants should internalise and acquire characteristics of that outgroup identity, lowering their prejudice towards that group after play (H1). We also hypothesised that the Proteus effect would prompt changes in empathy towards the outgroup, as empathic concern is a key component of prejudice reduction (H2). Finally, we wanted to examine what correspondence there may be between explicit and implicit expressions of prejudice (RQ1).

Overall, our hypotheses were supported. Participants who played with the avatar of the outgroup showed less explicit prejudice towards the outgroup following gameplay compared to the control condition (supporting H1). The experimental group also showed greater empathy towards the outgroup (supporting H2). Concerning RQ1, this pattern seemed to be present for implicit prejudice, suggesting that the Proteus effect may work on both forms of attitudes. Participants who played as a Syrian refugee showed smaller response latencies in incongruent trials, demonstrating lower prejudice. However, this effect was not significant and so a definitive conclusion cannot be drawn from these data.

The findings from this study support the notion that implicit prejudice is not simply the unconscious expression of explicit prejudice but is a more complex and potentially idiosyncratic construct. Implicit prejudice comes from long-term exposure to social situations and is more ingrained (Henry & Hardin, 2006; Wilson et al., 2000), which may mean it is harder to change.

### 4.1. Methodological Limitations and Future Work

An aspect of the Proteus effect experience is the contact participants have with a member of the outgroup through a representative avatar. Although contact can be effective at an implicit and explicit level (Vezzali & Giovannini, 2011), it is nevertheless important to account for individuals’ previous experiences with that outgroup when looking at effects. Previous negative interactions with an outgroup member can subsequently lead to more prejudicial attitudes (Meleady et al., 2017). Moreover, this effect can occur even if an individual has no direct contact with the outgroup, and instead comes from negative experiences of a friend with that group (Lutterbach & Beelmann, 2023), from media (Lynott et al., 2019), or through the subtle effects of cultural transmission (Pettigrew & Meertens, 1995). Negative contact experiences and their associated prejudice are more difficult to change (Aberson, 2015; Barlow et al., 2012; Kotzur & Wagner, 2021), and there are individual differences in personality, for instance, which may account for variations in bias. In this study, we did not ask participants about their previous experiences with the outgroup. This should ideally be included in follow-up research.

Explicit and implicit attitudes are also influenced by an individual’s desire to appear unbiased. Researchers (Dunton & Fazio, 1997) have developed and used a motivation to control prejudice scale and found it an accurate predictor of explicit attitudes. Participants with a strong motivation to appear impartial to outgroups have shown less explicit prejudice, while their implicit prejudice remained unaffected (Akrami & Ekehammar, 2005; Urbiola et al., 2017). However, subsequent research has found this motivation may also moderate implicit attitudes as well (Kurita & Kusumi, 2009; Schmader et al., 2011). This should also be accounted for when extending this work.

We may also want to examine the suitability of the IAT as a measure of implicit prejudice. Although seemingly robust and widely used, some researchers suggest that IAT scores may simply reflect familiarity or cultural associations, rather than attitudes the individual advocates (Bergh et al., 2012; Blanton & Jaccard, 2006). This may in part explain differing findings concerning implicit and explicit attitudes. Other studies have used a pseudo-implicit qualitative text measure of prejudice (Stiff & Kedra, 2020), which may help bolster IAT findings. Finally, we may also want to consider other individual differences in future work. Certain personality variables such as openness to experience (Akrami & Ekehammar, 2005) or social dominance orientation (Sibley & Duckitt, 2008) can moderate explicit prejudice, which may be important when considering the Proteus effect in subsequent studies (Fiske, 2000).

### 4.2. Conclusions

In this paper, we have demonstrated the effectiveness of asking individuals to take on the identity of an outgroup member in reducing prejudice towards that outgroup—the Proteus effect. This research extends our current understanding of the Proteus effect, which has previously focused on eliciting stereotypical attitudes and behaviour congruent with the acquired identity. Here, we have shown how the Proteus effect can cause positive change by reducing prejudice. Moreover, this effect has also extended to empathic concern—a key concept in prejudice reduction—and seemed to impact implicit prejudice, although this was not significant.

With this initial finding in place, we aim to expand on this idea in future work, incorporating key individual differences—such as prior outgroup experiences—into our understanding. With the continued popularity of video games, this research offers an optimistic view of how we may use this form of recreation for the betterment of society.

## Data Availability

The data presented in this study are available on request from the corresponding author due to privacy reasons.
